# Expression of concern: Comparing the predictive value of quantitative magnetic resonance imaging parametric response mapping and conventional perfusion magnetic resonance imaging for clinical outcomes in patients with chronic ischemic stroke

**DOI:** 10.3389/fnins.2023.1354585

**Published:** 2024-01-04

**Authors:** Frontiers Editorial Office

With this notice, Frontiers acknowledges concerns received by the office regarding data ownership and integrity of the article “Comparing the predictive value of quantitative magnetic resonance imaging parametric response mapping and conventional perfusion magnetic resonance imaging for clinical outcomes in patients with chronic ischemic stroke” published on 25 May 2023. In accordance with COPE guidelines, Frontiers has requested an investigation by Shanghai Jia Tong University and Grenoble University. Once the outcome of this investigation is made available to Frontiers, we will act accordingly.

UPDATE: This article has now been retracted. Please find the full retraction here: https://doi.org/10.3389/fnins.2024.1504798

